# Circumcision of Male Children for Reduction of Future Risk for HIV: Acceptability among HIV Serodiscordant Couples in Kampala, Uganda

**DOI:** 10.1371/journal.pone.0022254

**Published:** 2011-07-20

**Authors:** Kenneth K. Mugwanya, Christopher Whalen, Connie Celum, Edith Nakku-Joloba, Elly Katabira, Jared M. Baeten

**Affiliations:** 1 Department of Epidemiology and Biostatistics, School of Medicine, Case Western Reserve University, Cleveland, Ohio, United States of America; 2 Infectious Diseases Institute, School of Medicine, Makerere University, Kampala, Uganda; 3 Department of Epidemiology and Biostatistics, College of Public Health, University of Georgia, Athens, Georgia, United States of America; 4 Department of Epidemiology and Biostatistics, School of Public Health, Makerere University, Kampala, Uganda; 5 Departments of Global Health, Medicine, and Epidemiology, University of Washington, Seattle, Washington, United States of America; 6 Department of Epidemiology and Biostatistics, School of Public Health, Makerere University, Kampala, Uganda; 7 Department of Medicine, School of Medicine, Makerere University, Kampala, Uganda; Tulane University, United States of America

## Abstract

**Introduction:**

The ultimate success of medical male circumcision for HIV prevention may depend on targeting male infants and children as well as adults, in order to maximally reduce new HIV infections into the future.

**Methods:**

We conducted a cross-sectional study among heterosexual HIV serodiscordant couples (a population at high risk for HIV transmission) attending a research clinic in Kampala, Uganda on perceptions and attitudes about medical circumcision for male children for HIV prevention. Correlates of willingness to circumcise male children were assessed using generalized estimating equations methods.

**Results:**

318 HIV serodiscordant couples were interviewed, 51.3% in which the female partner was HIV uninfected. Most couples were married and cohabiting, and almost 50% had at least one uncircumcised male child of ≤18 years of age. Overall, 90.2% of male partners and 94.6% of female partners expressed interest in medical circumcision for their male children for reduction of future risk for HIV infection, including 79.9% of men and 87.6% of women who had an uncircumcised male child. Among both men and women, those who were knowledgeable that circumcision reduces men's risk for HIV (adjusted prevalence ratio [APR] 1.34 and 1.14) and those who had discussed the HIV prevention effects of medical circumcision with their partner (APR 1.08 and 1.07) were significantly (p≤0.05) more likely to be interested in male child circumcision for HIV prevention. Among men, those who were circumcised (APR 1.09, p = 0.004) and those who were HIV seropositive (APR 1.09, p = 0.03) were also more likely to be interested in child circumcision for HIV prevention.

**Conclusions:**

A high proportion of men and women in Ugandan heterosexual HIV serodiscordant partnerships were willing to have their male children circumcised for eventual HIV prevention benefits. Engaging both parents may increase interest in medical male circumcision for HIV prevention.

## Introduction

WHO/UNAIDS recommends medical male circumcision as part of a comprehensive HIV prevention package in countries with high HIV prevalence where circumcision is uncommon [1], following clinical trial data demonstrating that circumcision of adult men reduces HIV-1 risk by ∼60% [2], [3], [4]. Male circumcision programs for adults have subsequently been initiated in a number of African countries.

The ultimate success of medical male circumcision for maximizing HIV prevention may depend on circumcising male infants and children before they reach adolescence, in addition to adults. Infant circumcision is technically easier than the adult procedure, can be conducted shortly after birth, carries a low risk of complications, and may be cost-effective, even in regions with low HIV prevalence [5]. Thus, infant circumcision provides an important option for scaling up male circumcision for long-term HIV prevention at the individual and population levels. Perceptions about medical circumcision of male children specifically for reduction of future risk for HIV acquisition have been largely unexplored, although understanding parental preferences related to child circumcision may facilitate successful implementation of medical male circumcision [6]. Previous acceptability studies have demonstrated high interest in infant circumcision [7], [8], but most of these studies were conducted prior to the completion of the randomized trials demonstrating the efficacy of circumcision for HIV prevention [2], [3], [4].

We examined perceptions and attitudes of men and women in heterosexual HIV serodiscordant relationships about infant medical male circumcision for HIV prevention. We chose heterosexual serodiscordant couples because this group is an important population for HIV prevention, has been exposed to HIV prevention messages, and provides both male and female perspectives about potential male circumcision for their children.

## Methods

As previous described [9], [10], [11], between May and August 2008 heterosexual HIV serodiscordant couples attending a research clinic in Kampala, Uganda were interviewed separately by a gender-matched research assistant using a standard questionnaire. The primary aim of this cross-sectional survey was to assess knowledge and perceptions about adult male circumcision for HIV prevention [10]. A secondary goal of the study was to assess willingness to circumcise male children for potential reduction of future risk for HIV infection. The preferred age for circumcision of male child was assessed by an open ended question: “At what age would you prefer your male child to get circumcised **ONLY** for purposes of preventing HIV in the future?”. The project was conducted prior to release of a Uganda national policy on male circumcision [12] but after completion and dissemination of results from the randomized trials of circumcision for HIV prevention in men [2], [3], [4], one of which was done in Uganda [4]. Participant's HIV status was abstracted from their medical records, and the male partner's circumcision status was evaluated by physical exam. The institutional review boards of Case Western Reserve University, the University of Washington, and the Uganda National Council of Science and Technology approved the study. Participants provided written informed consent.

Continuous variables were summarized by median with interquartile range (IQR) and proportions for categorical variables. We assessed correlates of willingness to circumcise male children using generalized estimating equations methods with a Poisson link to generate prevalence ratios and 95% confidence intervals [13]. Men and women were analyzed separately. Variables that had a p-value≤0.2 in univariate analysis were included in multivariable models.

## Results

### General Characteristics

318 HIV serodiscordant couples were interviewed, 163 (51.3%) in which the female partner was the HIV uninfected member. The median age was 37 years (IQR, 31–43) for men and 31 years (IQR, 25–37) for women. Most couples were married and cohabiting. Almost half of males (53.8%, n = 171) of males and one third (36.5%, n = 116) of females had attained at least secondary level education. Religious distribution of this study population closely mirrored that of Uganda [14] : 84% were Christian and 16% were Muslim.

Ninety-nine (31.2%) men were circumcised, 5 (1.6%) were partially circumcised (i.e., with glans partially covered), and 213 (67.2%) were uncircumcised; one man declined examination to verify circumcision status, but was included in the analyses based on self-reported circumcision status. Nearly 50% (149/318) of couples had at least one uncircumcised male child≤18 years of age.

### Attitudes towards infant circumcision

Overall, 90.2% (285/316) of male partners (91.9% HIV positive / 88.4% HIV negative) and 94.6% (300/317) of female partners (95.5% HIV positive / 93.8% HIV negative) expressed interest in circumcision for their male children for reduction of future risk for HIV infection. When the analysis was restricted to the 149 partnerships with uncircumcised male children≤18 years, 79.9% of men (83.0% HIV positive / 74.6% HIV negative) and 87.6% of women (89.1% HIV positive / 86.7% HIV negative) expressed interest in circumcision of their male children. There was high within-couple agreement about interest in circumcision of their male children (percent agreement = 87.3% [276/316], 95% CI 83.0–90.4). In partnerships who disagreed on circumcision of male children (n = 40), female partners were more likely to support infant circumcision compared to their male partners (matched pair odds ratio = 2.10, 95% CI 1.03–4.38).

### Correlates of willingness to circumcise male children for reduction of future risk for HIV

Among both men and women, those who were knowledgeable that circumcision reduces adult men's risk for becoming HIV infected (adjusted prevalence ratio [APR] = 1.34 for men and APR = 1.14 for women) and those who had discussed circumcision with their partner (APR = 1.08 for men, and APR = 1.08 for women) were significantly more likely to be interested in male child circumcision for HIV prevention (Table 1). In men, those who were circumcised (APR = 1.09) and those who were HIV seropositive (APR = 1.09) were more likely to be interested in male child circumcision for HIV prevention.

**Table 1:** Correlates of willingness to circumcise male children for reduction of future risk for HIV.

	Male partners (N = 316)			Female partners (N = 317)		
Characteristics	Willing to circumcise male childn/total (%)*	A PR† (95% CI)	P-value	Willing to circumcise male childn/total (%)*	A PR† (95% CI)	P-value
**Age (years)**						
<30	56/61 (91.8)	-	-	135/142 (95.7)	-	-
30–39	122/133 (91.7)	-	-	114/121 (94.2)	-	-
≥40	107/122 (87.7)	-	-	51/54 (94.4)	-	-
**HIV Status**						
Positive	148/161 (91.9)	**1.09 (1.01, 1.18)**	**0.03**	148/155 (95.5)	-	-
Negative	137/155 (88.4)	Reference		152/162 (93.8)	-	-
**Male partner's circumcision status**						
Circumcised	93/95 (97.9)	**1.09 (1.02, 1.16)**	**0.004**	110/113 (97.4)	-	-
Uncircumcised	192/221 (86.9)	Reference		190/204 (93.1)	-	-
**Residence**						
urban	203/220 (92.3)	-	-	208/218 (95.4)	-	-
Rural	82/96 (85.4)	-	-	92/99 (92.9)	-	-
**Education level attained**						
≤Primary	128/145 (88.3)	-	-	190/201 (94.5)	-	-
≥Secondary	157/171 (91.8)	-	-	110/116 (94.8)	-	-
**Prior discussion about circumcision with partner**						
Yes	75/76 (98.7)	**1.08 (1.02, 1.14)**	**0.009**	84/84 (100)	**1.07 (1.03, 1.10)**	**<0.001**
No	210/240 (87.5)	Reference		216/233 (92.7)	Reference	
**Aware that circumcision reduces risk of HIV infection in adult men**						
Yes	236/243 (97.1)	**1.34 (1.20, 1.49)**	**<0.001**	273/284 (96.1)	**1.14 (1.00, 1.30)**	**0.05**
No	49/73 (67.1)	Reference		27/33 (81.8)	Reference	
**Male child<18 years with partner**						
Yes	164/186 (88.2)	-	-	177/186 (95.2)	-	-
No	121/130 (93.1)	-	-	123/131 (93.9)	-	-
**Religion**						
Christian	230/260 (88.5)	-	-	252/267 (94.4)	-	-
Muslim	55/56 (98.2)	-	-	48/50 (96.0)	-	-

*Row percentages displayed; APR adjusted prevalence ratios.

† Addition of age, religion, education level, and place of residence did not substantially affect estimates from the multivariable models.

Notably, we did not find significant statistical associations between age, having male children≤18 years, place of residence, education level attained, religious affiliation, place of residence, current employment status, and ethnicity with interest in infant male circumcision for future HIV prevention among either men or women.

### Preferred age for circumcision

**Figure 1:**
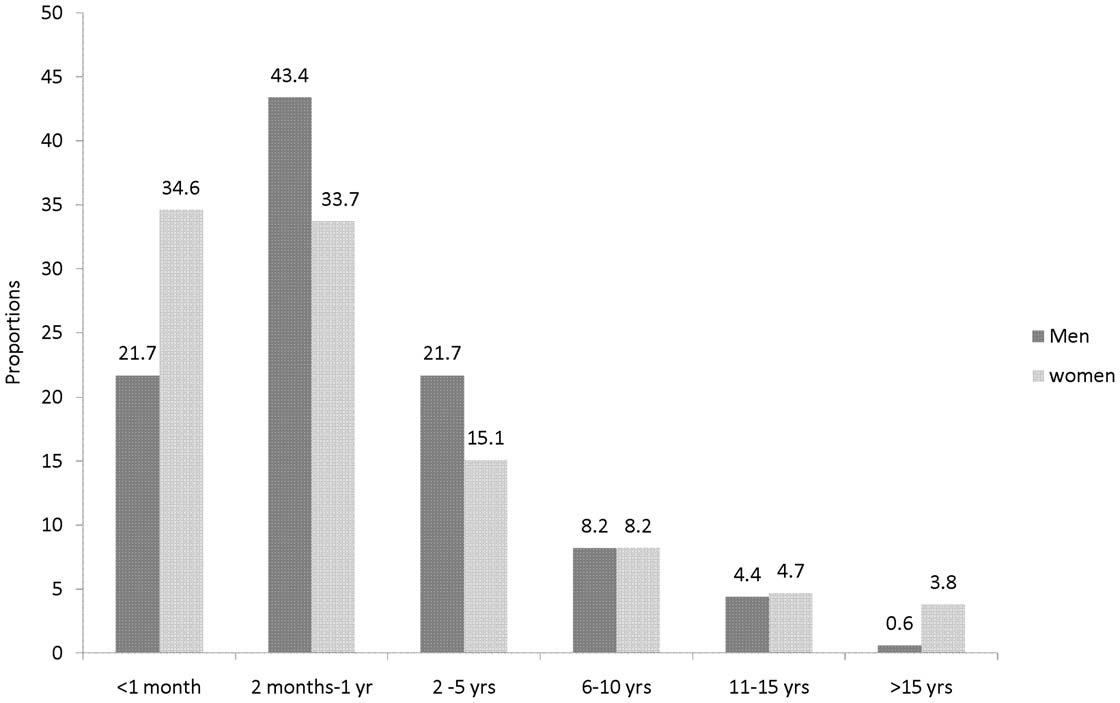
Preferred age for medical circumcision of male children stratified by the parent's gender.

Among those who favored circumcision, the median preferred age for circumcision of male children for purposes of future reduction of HIV risk was 6 months [IQR 1–36] among male partners and 2 months [IQR 1–36] for female partners (Figure 1). More than 60% of both men and women favored circumcision of children as infants (i.e., ≤12 months of age).

## Discussion

Over 90% of men and women in HIV serodiscordant partnerships in Kampala were interested in medical circumcision of male children to reduce future risk for HIV, including over 80% of those who had uncircumcised male child≤18 years of age. Couples indicated a preference for circumcision of male children as an infant rather than at an older age. These findings indicate high acceptability of medical male circumcision for infants and children of persons in heterosexual HIV serodiscordant couples, who are themselves a priority population for HIV prevention and may be especially motivated to find prevention strategies for their children.

Our results are consistent with positive attitudes towards adult male circumcision measured in our study population, in which we found that 53.3% of men and 88.1% of female partners in couples with an uncircumcised HIV seronegative male partner expressed interest in adult medical male circumcision [10]. Notably, we also found that HIV positive males were more likely to express interest in circumcision of their male children than HIV uninfected males, perhaps because of greater awareness of HIV risk. In general, women were more likely to favor circumcision for children than men, thus highlighting the opportunity to include both partners in counseling about circumcision of their male children. Our results are consistent with a study from Botswana that found a majority of postpartum mothers preferred circumcision of their male children before their first birthday [15]; however, that study did not obtain preference information from men about circumcision of their male infants. Similarly, these data are also consistent with findings from studies of parents regarding circumcision of their male children from populations other than sub-Saharan Africa [16], [17], [18].

In Africa, male circumcision is primarily for religious or cultural reasons, with many circumcising communities performing the procedure as a rite of passage to adulthood. For non-circumcising communities, which is characteristic of the majority of Uganda, neonatal circumcision could potentially be implemented easily, since there's no cultural connotation for the timing of circumcision. Our data are supportive of this concept, in that we found that the majority of men and women supported male circumcision in infancy for HIV prevention. In contrast to findings from a study among mothers in India [18], religion did not appear to influence willingness to circumcise male children in our study population; in different populations, perceptions about circumcision for religious reasons versus circumcision for medical purposes may be important for anticipating interest in child circumcision. In our study, the questionnaire was designed to assess interest in male child circumcision specifically for purposes of reduction of future risk for HIV. Of note, our data were collected prior to the recently-released policy in Uganda regarding male circumcision scale-up [12], which includes implementing infant and adult male circumcision. With several African countries in various stages of large scale roll-out of circumcision programs for HIV prevention, counseling messages to parents should provide balanced information about short-term benefits (reduced urinary tract infections), hygiene [19], and long-term benefits after adolescence with reduced susceptibility to HIV infection.

One limitation of this study is cross-sectional design and non-probability sampling. The study population included primarily (76%) research experienced HIV serodiscordant couples and may not be representative of couples who are unaware of their HIV status. We did not see significant differences in attitudes between couples in follow up and first time clinic attendees. Paradoxically, the strong effect we saw in a largely research experienced study population reinforces that knowledge of HIV prevention strategies in general could facilitate uptake of infant medical circumcision. We did not collect data on whether couples wanted to have additional children or how many of their current male children were infants versus older children. Future studies may be needed to assess if our findings are generalizable to HIV seroconcordant couples, including couples in which both members are HIV seronegative and couples in which both members are HIV seropositive.In conclusion, our data add to the increasing evidence in support of considering infant medical male circumcision as part of HIV prevention efforts and underscore the opportunity to engage men and women in discussion of circumcision for HIV prevention.

## References

[1] WHO/UNAIDS (28 March, 2007). http://www.who.int/mediacentre/news/releases/2007/pr10/en/index.html.

[2] Auvert B, Taljaard D, Lagarde E, Sobngwi-Tambekou Jl, Sitta Rm (2005). Randomized, controlled intervention trial of male circumcision for reduction of HIV infection risk: the ANRS 1265 Trial.. PLoS Medicine.

[3] Bailey RC, Moses S, Parker CB, Agot K, Maclean I (2007). Male circumcision for HIV prevention in young men in Kisumu, Kenya: a randomised controlled trial.. Lancet.

[4] Gray RH, Kigozi G, Serwadda D, Makumbi F, Watya S (2007). Male circumcision for HIV prevention in men in Rakai, Uganda: a randomised trial.. Lancet.

[5] Binagwaho A, Pegurri E, Muita J, Bertozzi S (2010). Male circumcision at different ages in Rwanda: a cost-effectiveness study.. PLoS Med.

[6] Kalichman SC (2010). Neonatal circumcision for HIV prevention: Cost, culture, and behavioral considerations.. PLoS Med.

[7] Lagarde E, Dirk T, Puren A, Reathe RT, Bertran A (2003). Acceptability of male circumcision as a tool for preventing HIV infection in a highly infected community in South Africa.. AIDS.

[8] Mattson CL, Bailey RC, Muga R, Poulussen R, Onyango T (2005). Acceptability of male circumcision and predictors of circumcision preference among men and women in Nyanza Province, Kenya.. AIDS Care.

[9] Celum C, Wald A, Lingappa JR, Magaret AS, Wang RS (2010). Acyclovir and transmission of HIV-1 from persons infected with HIV-1 and HSV-2.. N Engl J Med.

[10] Mugwanya KK, Baeten JM, Nakku-Joloba E, Katabira E, Celum C (2010). Knowledge and attitudes about male circumcision for HIV-1 prevention among heterosexual HIV-1 serodiscordant partnerships in Kampala, Uganda.. AIDS Behav.

[11] Lingappa JR, Lambdin B, Bukusi EA, Ngure K, Kavuma L (2008). Regional differences in prevalence of HIV-1 discordance in Africa and enrollment of HIV-1 discordant couples into an HIV-1 prevention trial.. PLoS One.

[12] Wairagala W (2010). Uganda steps up efforts to boost male circumcision.. Lancet.

[13] Petersen MR, Deddens JA (2008). A comparison of two methods for estimating prevalence ratios.. BMC Med Res Methodol.

[14] MOH Uganda HIV / AIDS Sero-Behavioural Survey, 2004–2005: Available at: http://www.measuredhs.com/pubs/pdf/AIS2/AIS2.pdf. Accessed March 20, 2011

[15] Plank RM, Makhema J, Kebaabetswe P, Hussein F, Lesetedi C (2009). Acceptability of Infant Male Circumcision as Part of HIV Prevention and Male Reproductive Health Efforts in Gaborone, Botswana, and Surrounding Areas.. AIDS Behav.

[16] Ahaghotu C, Okafor H, Igiehon E, Gray E (2009). Psychosocial factors influence parental decision for circumcision in pediatric males of African American descent.. J Natl Med Assoc.

[17] Castro JG, Jones DL, Lopez MR, Deeb K, Barradas I (2010). Acceptability of neonatal circumcision by Hispanics in southern Florida.. Int J STD AIDS.

[18] Madhivanan P, Krupp K, Chandrasekaran V, Karat SC, Reingold AL (2008). Acceptability of male circumcision among mothers with male children in Mysore, India.. AIDS.

[19] Singh-Grewal D, Macdessi J, Craig J (2005). Circumcision for the prevention of urinary tract infection in boys: a systematic review of randomised trials and observational studies.. Arch Dis Child.

